# Time series of cases and treatment outcomes from tuberculosis in Sergipe, 2012–2021

**DOI:** 10.1590/1980-549720230041

**Published:** 2023-09-18

**Authors:** Jefferson Felipe Calazans Batista, Vitória Steffany de Oliveira Santos, Carla Viviane Freitas de Jesus, Sonia Oliveira Lima

**Affiliations:** I Universidade Tiradentes – Aracaju (SE), Brazil.

**Keywords:** Tuberculosis, Time series studies, Seasons, Epidemiology, Tuberculose, Estudos de séries temporais, Sazonalidade, Epidemiologia

## Abstract

**Objective:**

The objectives of this study were: (1) to analyze the temporal trend of tuberculosis treatment outcomes in the state of Sergipe; (2) to identify the existence of seasonality of tuberculosis; (3) to verify the influence of the rapid molecular test (MTB-RIF) in the time series of tuberculosis and its treatment outcomes in the state of Sergipe; and (4) to verify treatment outcomes.

**Methods:**

Ecological study on tuberculosis and three treatment outcomes (cure, interruption of treatment, and death) extracted from Datasus. Incidence and mortality rates were calculated for the crude occurrences of cases and deaths and the proportions of cure and interruption of treatment (%). The time series was analyzed using Prais-Winsten regression from Jan to Dec/2021.

**Results:**

The total incidence rate was 36.35 cases per 100,000 inhabitants, with an increase of 0.44% per month (95%CI 0.35; 0.54). The cure rate was 64.0% with a steady trend (p>0.05). The percentage of treatment interruption was 13.3%, with a reduction of −0.73%/month (95%CI −1.11; −0.34). The total mortality rate was 1.92 deaths/100,000 inhabitants with a stationary trend. After the implementation of the MTB-RIF, there was an increase in the incidence rate of 0.65% per month. Seasonality was not identified in any of the analyses performed (p>0.05).

**Conclusion:**

There was an increase in incidence rates, reduction in treatment interruption and mortality in the state of Sergipe. Seasonality was not identified. The rapid molecular test showed a growth effect on the incidence rate.

## RESUMO

**Objetivo:**

Os objetivos deste estudo são, no estado de Sergipe: (1) analisar a tendência temporal da tuberculose e os desfechos do tratamento; (2) identificar a existência de sazonalidade da tuberculose; (3) verificar a influência do teste rápido molecular (TRM-TB) na série temporal da tuberculose e seus desfechos de tratamento; e (4) verificar os desfechos do tratamento.

**Métodos:**

Estudo ecológico sobre tuberculose e três desfechos do tratamento (cura, interrupção do tratamento e óbito) extraídos do Datasus. Foram calculadas as taxas de incidência e de mortalidade para as ocorrências brutas de casos e as proporções de cura e interrupção do tratamento (%). A série temporal foi analisada pela regressão de Prais-Winsten no período de janeiro de 2012 a dezembro de 2021.

**Resultados:**

A taxa de incidência total foi de 36,35 casos por 100 mil habitantes, com aumento de 0,44% ao mês (IC95% 0,35; 0,54). A proporção de cura foi de 64,0%, com tendência estacionária (p>0,05). O percentual de interrupção do tratamento foi de 13,3%, com redução de 0,73% ao mês (IC95% −1,11; −0,34). A taxa de mortalidade total foi de 1,92 morte por100 mil habitantes, com tendência estacionária. Posteriormente à implementação do TRM-TB, a taxa de incidência cresceu 0,65% ao mês. A sazonalidade não foi identificada em nenhuma das análises realizadas (p>0,05).

**Conclusão:**

Cresceram as taxas de incidência, redução da interrupção do tratamento e mortalidade no estado de Sergipe. Não foi identificado sazonalidade. O teste rápido molecular apresentou efeito de crescimento na taxa de incidência.

## INTRODUCTION

Tuberculosis, an infectious and contagious disease, is caused by Mycobacterium tuberculosis (Mtb)^1,2^ and implies social, economic and health losses, as it can result in temporary or permanent disability, resulting from the disease process itself or from side effects related to the treatment, primarily to second-line drugs used to contain drug-resistant tuberculosis^3^.

Tuberculosis is a global health problem, which generates social, economic, and health impacts. Economic, social, and environmental factors can influence the burden of tuberculosis in a given population^2,4,5^.

Over the past few years, numerous actions have emerged to control tuberculosis, including the Rapid Test Network for Tuberculosis, which defined the municipalities in which the rapid molecular test for tuberculosis (MTB-RIF)^6^ would be implemented in 2014 and 2015. It uses a test based on the Genexpert MTB-RIF method, which is automated, simple, fast, and easy to perform — it is capable of detecting Mtb and indicating resistance to rifampicin in two hours on average^6^.

The institution of this tool in the Brazilian Unified Health System (*Sistema Único de Saúde* – SUS) was a great advance for the early detection of cases, which can impact the indicators of adequate treatment and reduce the interruption of treatment, as well as the number of deaths^7,8^.

Brazil is one of the 30 countries with the highest burden of tuberculosis listed by the World Health Organization (WHO)^4^. This burden has been considered a priority in Brazilian public policies since 2003^9^. From 2006 to 2015, the incidence rate of tuberculosis was 36.8 cases/100,000 inhabitants^10^. Among the macro-regions, the Northeast had the third highest incidence rate (36.2/100,000 inhabitants) and the highest mortality rate (2.9 deaths per 100,000 inhabitants)^10^.

Sergipe has one of the lowest average incidence rates in the Northeast (34.6/100,000), however, the literature shows that the state has spatial dependence and high-risk areas for the disease^11–13^.

Given the above, the objectives of this study were, in the state of Sergipe:

1. To analyze the time trend of tuberculosis and treatment outcomes;
2. To identify the existence of seasonality of the condition;
3. To verify the influence of the rapid molecular test (MTB-RIF) in the time series of tuberculosis and its treatment outcomes; and
4. To evaluate treatment outcomes.

## METHODS

Quantitative, exploratory, and analytical time series ecological study, which used data from tuberculosis cases and three treatment outcomes (cure, interruption of treatment, and death from tuberculosis) selected due to being considered important epidemiological indicators for disease control.

Data on cases, cure, and interruption of treatment were extracted from the Information System for Notifiable Diseases (*Sistema de Informação de Agravos de Notificação* – Sinan), while deaths came from the Information System on Mortality (*Sistema de Informação sobre Mortalidade* – SIM), classified with ICD-10 code A15 to A19. All data are available at the Department of Informatics of the Unified Health System (*Departamento de Informática do Sistema Único de Saúde* – Datasus) and were accessed via Tabnet on October 15^th^, 2022.

The selected geographic level was the state of Sergipe, located in the Northeast Region, which occupies a total area of 21,910 km^2^ and has an estimated population, in 2022, of 2.3 million inhabitants (107.76 people/km^2^). The state is divided into seven health regions (HR): Aracaju, Estância, Itabaiana, Lagarto, Nossa Senhora da Glória, Nossa Senhora do Socorro, and Propriá^14^.

The selected variables were Federation Unit (Sergipe) and HR of residence, month/year of diagnosis (from January 2012 to December 2021) and termination situation (cure, interruption of treatment, and death from tuberculosis). The other termination situations (death from other causes, transfer, change in diagnosis, drug-resistant tuberculosis (DRTB), and change in regimen) were removed as they were not part of the scope of this investigation. Skipped or blank data have been removed.

Data were analyzed using descriptive statistics: absolute (N) and relative (%) frequency. Incidence and mortality rates were calculated for the crude occurrences of cases and deaths, using the following formula:


$$\frac{o_{i}}{p_{i}}\times 100,000$$

Where:

o_i_: cases or deaths from tuberculosis in a given place and period; and

p_i_: population residing in the same place and period.

Population estimates for Sergipe and its HR come from intercensal estimates by the Brazilian Institute of Geography and Statistics (*Instituto Brasileiro de Geografia e Estatística* – IBGE) from 2012 to 2021^15^.

The crude occurrences of cure and interruption of treatment were expressed by means of proportions, dividing the observed values of cure and interruption of treatment by the total cases at the same place and time.

For the analysis of time series in the present study, the linear regression model with Prais-Winsten autocorrelation correction was used^16^.

The calculations used considered time (month/year) as an independent variable and incidence and mortality rates as dependent variables, as well as the proportion of cure and interruption of treatment. All indicators were transformed into a base 10 logarithm, which allows reducing the heterogeneity of the residual variance and correcting deviations from normality. Based on the regression results, the percentage variation and its respective 95% confidence interval (95%CI) were estimated using the formulas^16^:


$$\begin{array}{l} Percent\;variation=[-1+10^{b1}]\times 100\%\\ 95\%CI_{minimum}=\left[-1+10^{CI\;min.\;of\;b1}\right]\times 100\%\\ 95\%CI_{maximum}=\left[-1+10^{CI\;max.\;of\;b1}\right]\times 100\% \end{array}$$

This indicator is used to describe and quantify the trend. Negative results indicate a decrease in records, while positive results indicate an increase, and when there is no statistical significance (p>0.05), it is a stationary trend^16^.

It should be noted that the calculations of the trend for the crossing of the closure status variable by health region were performed per year due to the large proportion of zero values. It should also be noted that, in time series, there is the problem of serial autocorrelation, which can bias the interpretation of the regression result, which is why the Durbin-Watson estimate was used to support adequate interpretations — values between 1.5 and 2.5 were considered reliable^17,18^.

In order to identify the seasonal behavior of incidence and mortality rates and the proportion of cure and interruption of treatment, the model described by Antunes and Cardoso^16^ was adopted. For seasonality modeling, the time series was decomposed in order to isolate the seasonal component. Decomposition uses the linear regression formula with two new components:


$$Y=b_{0}+b_{1}X_{i}+b_{2}*sin\left(\frac{2\pi X_{i}}{L}\right)+b_{3}*\cos\left(\frac{2\pi X_{i}}{L}\right)$$

Where:

Y = coefficients;

*X_i_*: sequential time numbering;

π: constant (3.1415…);

L: way of measuring time; and

b_2_ (sine) and b_3_ (cosine): seasonality modelers.

In the present study, the coefficients are those previously mentioned; *X_i_* indicates the analyzed month and year; and L refers to the way in which time is measured, that is, monthly (12). As for the coefficients b_2_ (sine) and b_3_ (cosine), which model seasonality, if one or both are statistically significant, it is concluded that there is seasonal variation, otherwise, the variation is attributed to chance^16^.

For the analysis of interrupted time series, two new measures (level and trend) were inserted in the Prais-Winsten model, together with the time variable and the coefficients. The level assesses whether the intervention influenced an immediate change in the time series, for this purpose a variable categorized as 0 and 1 was created, which divides the period into two moments: before intervention (value 0) and after intervention (value 1).

The trend evaluates whether there was a gradual influence after the intervention, thus, a discrete variable was created, to which, the value 0 was assigned in the period prior to the implementation of the TRM-TB, and after the implementation, the value 1, with gradual growth until the end of the time series (1, 2, 3, 4…).

These parameters, when positive and significant (p<0.05), indicate an immediate or gradual increase in the post-intervention outcome variable; however, when negative, they are interpreted as a decrease, and when they are not significant, they indicate that the event did not influences the time series^16^.

The unit used in this study was the MTB-RIF, applied for the first time in Sergipe in May 2015^19^.

For trend calculations, the Stata 16 program was used, for descriptive analysis and calculation of rate-type measures, Microsoft Excel 2019. The significance level of 5% (p<0.05) was adopted.

Appraisal by the Ethics and Research Committee was waived because it is a study with secondary data and public access.

## RESULTS

In the state of Sergipe, from January 2012 to December 2021, 8,167 cases of tuberculosis were reported, of which 5,225 progressed to cure, 1,085 patients discontinued treatment, and 432 died due to the aggravation of the disease. The incidence rate was 36.35 cases per 100,000 inhabitants.

**Figure 1:**
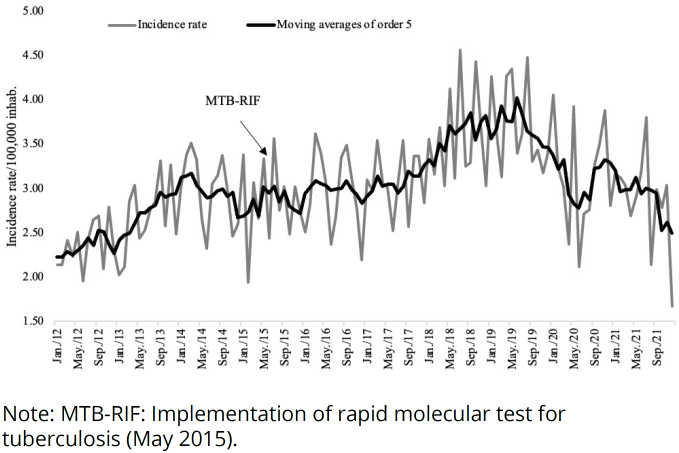
Time series of the incidence rate of tuberculosis (analyzed by moving averages of order 5) in the state of Sergipe, from January 2012 to December 2021.

Source: Research data, 2022.

In the visual inspection of the time series, it is possible to notice an increase in incidence from January 2012 to June 2019, with a subsequent decrease (Figure 1). The Prais-Winsten regression of the incidence rate increased by 0.44% (95%CI 0.35; 0.54, p<0.001) per month, until June 2019, since when a 1% reduction (95%CI - 1.62; −0.38, p=0.003) per month was registered.

The regression model did not show significant seasonality (cosine and sine: p>0.05). The implementation of MTB-RIF showed a statistically significant effect on the time series of the incidence rate (b=-0.00320; 95%CI −0.00529; −0.0010, p=0.003) (Figure 1). The intervention trend until the end of the series is stationary (p>0.05), however, until June 2019, it grew 0.65% per month (95%CI 0.41; 0.89).

The incidence rate increased in the regions of Aracaju, Nossa Senhora do Socorro, and Itabaiana, surpassing the month-over-month percent change (MPC) in the state, led by Nossa Senhora do Socorro (Table 1). In the other regions, it was stationary, however, analyzing the trend, disregarding the pandemic period, only the HR of Estância shows growth — 0.46% per month (95%CI 0.09; 0.82).

**Table 1:** Time trend of the incidence rate of tuberculosis in the state of Sergipe, in its health regions, from January 2012 to December 2021.

Location	MPC (%)	95%CI		D-W	Interpretation
		Lowest	Highest		
Sergipe	0.22*	0.10	0.33	1.945	Growing
HR Aracaju	0.32*	0.17	0.48	1.897	Growing
HR Estância	0.09	-0.18	0.36	1.914	Stationary
HR Lagarto	-0.04	-0.28	0.19	1.992	Stationary
HR N. S. Socorro	0.35*	0.10	0.61	1.955	Growing
HR Propriá	-0.07	-0.44	0.31	1.973	Stationary
HR N. S. Glória	-0.16	-0.44	0.12	1.984	Stationary
HR Itabaiana	0.31*	0.05	0.56	1.947	Growing

Source: Research data, 2022.

Note: MPC: month-over-month percentage change; 95%CI: MPC confidence interval; D-W: Durbin-Watson corrected by Prais-Winsten; HR: health region; N.S.: Nossa Senhora.

* p<0.05.

The regression model showed that there is no seasonal behavior of tuberculosis in the HRs (p>0.05). The implementation of the MTB-RIF had a significant effect on the incidence rate only in Estância and Lagarto, with a post-intervention percent change of −1.46% (95%CI −2.67; −0.23, p<0.05) and −1.40% (95%CI −2.42; −0.37, p<0.05) per month.

**Figure 2:**
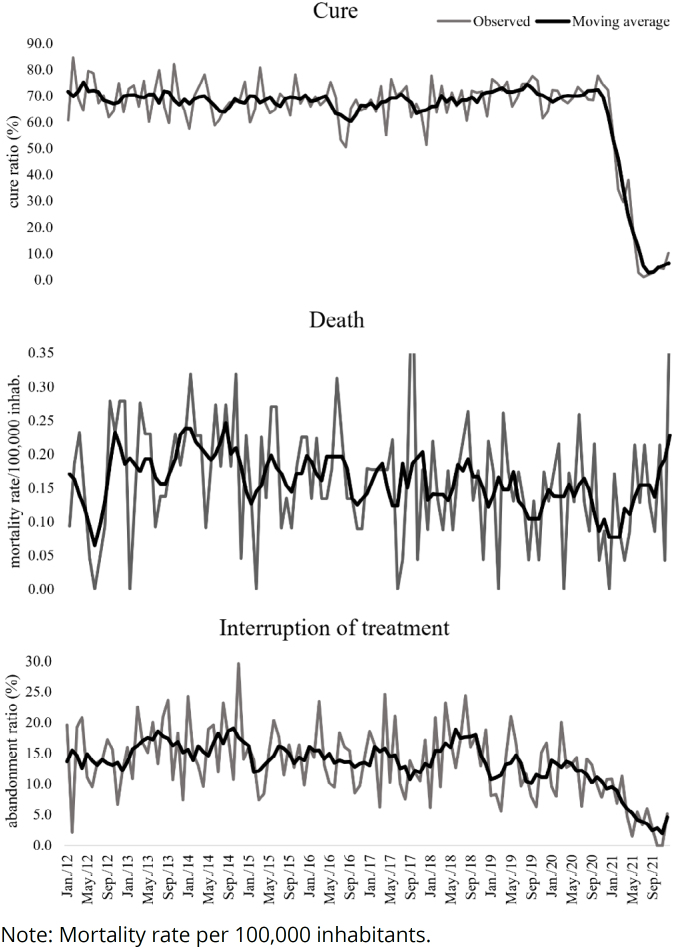
Time series of cure, treatment interruption, and mortality rate due to tuberculosis (analyzed by moving averages of order 5) in the state of Sergipe, from January 2012 to December 2021.

Source: Research data, 2022.

During the period, the cure rate in the state was 64.0%, recording an average of 63.7% (SD=17.6), a minimum of 1.1%, and a maximum of 84.8%. Visually, the series shows a stationary pattern until April 2020 and a declining rate (Figure 2) afterward. The Prais-Winsten model did not identify significant trend (p>0.05).

The proportion of treatment interruption in the state was 13.3%, with a mean of 13.3% (SD=5.8), a minimum of 0%, and a maximum of 29.6%. The temporal trend was a decrease of 0.73% per month (95%CI −1.11; −0.34, p<0.001).

The mortality rate from tuberculosis was 1.92 per 100,000 inhabitants and had a stationary pattern (p>0.05) (Figure 1). The influence of MTB-RIF, seasonality, and temporal trend were not identified in this time series (p>0.05).

With regard to HR, the highest proportion of cures was in Nossa Senhora da Glória, with 78.6%, followed by Estância (76.4%), Itabaiana (67.5%), Nossa Senhora do Socorro (66.4%), Lagarto (66.3%), Propriá (64.6%), and Aracaju (59.2%). The proportions of treatment interruption were: Nossa Senhora da Glória (5.1%), Estância (5.6%), Itabaiana (6.9%), Lagarto (8.6%), Propriá (12.0%), Nossa Senhora do Socorro (12.5%), and Aracaju (17.1%). Cure and interruption of treatment showed stationarity in their proportions. As for mortality, there was a reduction in the rate only in the HR of Nossa Senhora da Glória (Table 2). The seasonality and influence of MTB-RIF were not identified in the temporal analysis of health regions.

**Table 2:** Temporal trend of the proportion of cure and interruption of treatment and the mortality rate due to tuberculosis in the health regions of Sergipe, from 2012 to 2021.

Outcome	Health region	APC (%)	95%CI		D-W	Interpretation
			Lowest	Highest		
Cure	Aracaju	-6.79	-20.89	9.83	1.231	Stationary
	Estância	-8.96	-24.93	10.42	1.167	Stationary
	Lagarto	-9.93	-22.29	4.38	1.205	Stationary
	N. S. Socorro	-1.86	-13.45	11.29	1.213	Stationary
	Propriá	-12.94	-28.65	6.24	1.247	Stationary
	N. S. Glória	-8.70	-19.79	3.92	1.340	Stationary
	Itabaiana	-8.55	-23.92	9.93	1.253	Stationary
Interruption of treatment	Aracaju	-4.45	-16.70	9.61	1.328	Stationary
	Estância	-2.79	-10.95	6.11	1.930	Stationary
	Lagarto	-15.14	-30.13	3.06	1.453	Stationary
	N. S. Socorro	-14.52	-27.93	1.37	1.084	Stationary
	Propriá	-3.54	-25.63	25.11	1.266	Stationary
	N. S. Glória	-4.87	-17.22	9.34	1.751	Stationary
	Itabaiana	10.77	-22.84	3.18	1.596	Stationary
Death from TB	Aracaju	-5.00	-10.56	0.89	1.580	Stationary
	Estância	4.61	-7.33	18.09	1.792	Stationary
	Lagarto	-3.62	-14.07	8.11	1.953	Stationary
	N. S. Socorro	1.00	-4.28	6.57	1.925	Stationary
	N. S. Glória	-7.96*	-14.26	-1.19	2.006	*Decreasing*
	Propriá	1.15	-9.55	13.12	1.895	Stationary
	Itabaiana	-4.01	-15.96	9.64	1.862	Stationary

Source: Research data, 2022.

Note: APC: annual percent change; 95% CI: 95% confidence interval; D-W: Durbin-Watson corrected by Prais-Winsten; N.S.: Nossa Senhora; TB: tuberculosis.

* statistically significant at p<0.05.

## DISCUSSION

The present study made it possible to evaluate the temporal behavior of tuberculosis and its outcomes of cure, interruption of treatment, and death in the state of Sergipe. An increase in the incidence, stationarity of the proportions of cure, and reduction of the interruption of the treatment and of the deaths were identified. The implementation of MTB-RIF influenced the incidence rate of tuberculosis and there was no statistically significant seasonality.

The growing trend in the incidence of tuberculosis in the population of Sergipe contradicts the international^20,21^ and national^22–24^ literature, which indicates a decrease in the number of records. However, a study also carried out in the state, from 2001 to 2016, identified a stationary trend in incidence, with growth in some age groups and in males^25^.

The difference between the pattern observed in this study and that in the literature may have several reasons, including low adherence or applicability of public policies for tuberculosis control, whose measurement is extremely complex at the aggregate level and can be influenced by multiple factors.

It is noteworthy that Sergipe does not present favorable social indicators, highlighting it with a medium level of social vulnerability index (SVI)^26^. Furthermore, the state is among the only three federative units in the Northeast where there is no Tuberculosis Control Committee (TCC). It is a collegiate whose purpose is to act as a link between government and civil society, aiming to integrate these instances and contribute to public policies for tuberculosis control^27^.

Possibly, the absence of this collegiate would not be directly reflected in the number of cases, cure, interruption of treatment and death from tuberculosis in the state, but its presence would be a predictor for improving the indicators. It is worth noting that the Brazilian TCC Network was established in 2012^5^.

Another reason for this increase may be the intensification of screening and reporting of the disease. From 2012 to 2021, several public policies emerged, such as the Nursing Protocol for Directly Observed Treatment (DOT)^28^; recurring updates of the Manual of Recommendations for Tuberculosis Control in Brazil^29^; establishment of the Brazilian Network of State Committees for Tuberculosis Control, in 2012; Ordinance No. 1.271/2014^30^, which instituted the obligation of immediate notification of occurrences; implementation of the Molecular Rapid Test Network, in 2014; Joint Operational Instruction, which establishes general guidelines on tuberculosis in social assistance services; and creation of the Parliamentary Front of the Americas to fight the condition^5^. These policies were important to track and control the disease, which may explain the growth trend observed in Sergipe.

This research showed the significant influence of MTB-RIF on the incidence rate of tuberculosis after the period of its implementation — the coefficient grew by 0.65%.

This influence was also identified in a study carried out in Macapá (AP)^8^. This fact strengthens the hypothesis that this method facilitated and accelerated the detection of cases of this condition^31^. However, the importance of sputum smear microscopy, which remains the gold standard for diagnosing the disease, cannot be ruled out; its disadvantage is time (from 45 to 60 days, due to the slow replication of the bacillus^32^). Thus, the use of one or another therapy should not be isolated, but jointly, in order to eliminate the chances of false-positives and adequately screen resistant strains.

It was observed that mortality rates were stationary, except in the Nossa Senhora da Glória health region, which showed a reduction, a fact possibly related to the proportions of cure and interruption of treatment shown to be the lowest in the state. These findings corroborate the literature^20,33^, which points to poor adherence to treatment as one of the contributors to increased mortality rates from tuberculosis, especially when these rates are associated with unfavorable economic and educational indicators^24^.

A low proportion of treatment interruption results in adequate cure and reduces the chances of relapse, transmissibility, and death in the long term, considering that adequate treatment takes time. These factors also reinforce the idea that the diagnosis and notification of tuberculosis in Sergipe have been improving over the years, since case tracking allows DOT and a favorable outcome. The importance of drugs against DRTB and of secondary and tertiary health services to reduce death rate is also highlighted.

The seasonality evaluated in this study was not present in the estimated models. Some national surveys^8,34^ and in countries such as India^35^ and China^36^ have identified the presence of seasonality in the incidence rates of tuberculosis. Sergipe is situated, according to the classification of Koppen^37^, as having a hot climate with winter rain. The seasonal behavior can be attributed to rainy periods and lower temperature, which forces the collective to look for crowded spaces with little ventilation, allowing the dissemination of Koch's bacillus in the air. In times of hot weather, airy environments are usually more sought after, which decreases contagion.

However, for Paz et al.^34^, who evaluated the seasonality of tuberculosis in Brazilian capitals, including Sergipe (Aracaju), the climate may not be the main reason for the behavior of tuberculosis, since confounding factors tend to be present.

In the present study, it was observed that the incidence rates and proportion of cure and interruption of treatment decreased from 2019 until the end of the time series. It is known that, in January 2020, the COVID-19 pandemic arrived in Brazil and, in Sergipe, in March of that year, when the first cases were identified^38^. There are several factors that justify the reductions observed in the present study.

With regard to incidence, the reduction can be attributed to the process of diagnosis and notification of cases or to the delay in transmission of the bacillus. The pandemic generated intense impacts on health and care services, overloading different areas of care^39^. This overload may have limited the detection of tuberculosis cases, since the signs and symptoms of COVID-19 are similar to those of tuberculosis, making the differential diagnosis difficult^40,41^.

In addition, failures in the notification process may have occurred during this period, as shown by research on the impact of the pandemic on the diagnosis of tuberculosis in the state of Bahia, where the number of accumulated cases dropped by 26.4% from January to July 2020 compared to data from the same period of 2019^42^. On the other hand, measures such as social distancing and isolation, in addition to the use of a mask, may have contributed to this scenario, since both agents are transmitted by air^43^.

The proportion of cure and interruption of treatment in the population of Sergipe did not reach the limit recommended by the WHO, of 85.0 and 5.0%, respectively^44^, this represents approximately 2.6 times more interruptions in treatment and 1.3 times less outcomes of cure. The percentage of cure in the state is lower than the 70.4% found in Rio de Janeiro^45^ and the 70.0% in Minas Gerais^46^.

According to a survey carried out in all states, only in Acre and Piauí was the proportion of treatment interruptions lower than 5.0% from 2012 to 2018. This indicator in Sergipe was 11.8%, a rate similar to that identified in the present research^47^. This reality indicates that, even with the implementation of public policies, such as DOT, the interruption of treatment and, consequently, the cure still require corrective actions, since interruption rates are reflected in increased transmissibility, treatment costs, morbidity, and mortality, as well as drug resistance.

Because it is a secondary data study, this research has limitations, such as underreporting of cases. The influence of the pandemic period on the observed data may have limited statistical estimates, especially with regard to interrupted series, since despite being an unavoidable limitation, it is not a natural behavior of the disease. In addition, this research did not consider characteristics such as education, age group, and gender, which can be influenced by temporal and seasonal factors.

There was evidence of a growth pattern in the incidence rate of tuberculosis in the state of Sergipe and in the health regions of Aracaju, Itabaiana, and Nossa Senhora do Socorro. The proportion of cure and interruption of treatment did not reach what the WHO recommends. While the cure showed a stationary pattern, interruption of treatment and death registered a reduction. No significant seasonal pattern was identified, and the estimate of interrupted series indicated, after the implementation of the MTB-RIF, an increase in the incidence rate of tuberculosis in the state. In the period coinciding with the COVID-19 pandemic, data related to the incidence, the proportion of cures and the interruption of treatment throughout the Sergipe territory were reduced.

However, it is noteworthy that this study is a pioneer in the state, since only the health regions were considered and the seasonality and influence of MTB-RIF on cases and treatment outcomes were evaluated. Thus, the evidence presented may be important for directing actions and public policies to change the scenario of tuberculosis in Sergipe. In addition, this research can contribute to the achievement of the goals established in the National Plan to End Tuberculosis^5^ and the Sustainable Development Goals (SDG) 2030^48^.
